# Genome-wide CRISPR screen identified NEK6 as a determinant of sensitivity to CDK4/6 inhibitor in endometrial cancer

**DOI:** 10.3389/fphar.2025.1725886

**Published:** 2026-01-05

**Authors:** Fangfang Jian, Chongying Zhu, Yiwei Wang, Tianyu Zhou, Chenmin Yang, Weiwei Feng

**Affiliations:** Department of Obstetrics and Gynecology, Ruijin Hospital, Shanghai Jiao Tong University School of Medicine, Shanghai, China

**Keywords:** CDK4/6 inhibitor, endometrial cancer, genome-wide CRISPR screen, NEK6, YBX1

## Abstract

Endometrial cancer (EC) harbors highly recurrent cell cycle pathway alterations, especially hyperactivation of the CCND1/CDK4/6 axis, raising the potential for use of CDK4/6 inhibitors in these cancers. In this study, we performed a genome-wide CRISPR-Cas9-based knockout screen to identify genes that modify the response to CDK4/6 inhibitors. We found that NIMA-related kinase-6 (NEK6) levels determine the anti-tumor effects of CDK4/6 inhibitors and that NEK6 was a synthetic lethal target of CDK4/6 inhibitors in EC. We further demonstrated that combined inhibition of NEK6 and CDK4/6 resulted in marked suppression of tumor growth *in vitro* and *in vivo*. Mechanistically, NEK6 bound to Y-box binding protein-1 (YBX1) in the cytoplasm and facilitated its phosphorylation at serine 102 (p-YBX1^S102^), which promoted YBX1 nuclear translocation and further activated CDK2 and bcl2 transcription. As such, these data provided robust pre-clinical mechanistic evidence of synergy between NEK6 and CDK4/6 inhibitors and delineated a path for translation of these findings to preliminary clinical studies in EC patients.

## 1 Introduction

Endometrial cancer is the sixth most common cancer in women, with 417000 new diagnoses made globally in 2020 (Sung et al., 2021; Morice et al., 2016). In the past several years, the incidence of EC has increased according to the age and size of the population (Sung et al., 2021), with 132% increase over the last 30 years. Unfortunately, survival outcomes of women with EC have not improved over the past decades, and the most recent trends indicate a rise in mortality rates of 1.8% per year since 2010 (How et al., 2024). Immunotherapy has transformed the endometrial cancer treatment landscape, particularly for those exhibiting mismatch repair deficiency [MMRd/microsatellite instability-hypermutated (MSI-H)]. However, outcomes for patients with advanced-stage and metastatic endometrial cancer remain unsatisfactory. Thus, there is an urgent need to identify and develop more effective therapeutic strategies to improve the prognosis of EC.

Cell cycle is a series of tightly regulated molecular events that control cell division, and dysregulation of the cell cycle progression represents one of the hallmarks of tumor cells, thereby making cell cycle proteins an important target for anti-cancer therapy. Several studies have mapped this cell cycle-related genes derailments in the development and progression of EC.

Tsuda et al. investigated pRb/cyclin D1/p16INK4A/CDK4-pathway alterations in endometrioid-type endometrial carcinomas and hypothesized that aberrant expression of p16INK4A and CDK4 proteins constituted an early event during endometrial tumorigenesis (Semczuk and Jakowicki, 2004). In Tsuda’s study, the abnormalities of p16-cyclin D1/CDK4-pRb pathway were detected in 18 of 35 EC (51.4%) (Tsuda et al., 2000). In Semczuk’s study, half of the uterine carcinomas overexpressed cyclin D1 protein (Semczuk et al., 2004). These data raise clear hypotheses regarding targeting cyclin D-CDK4/6 in EC. Inhibitors of CDK4/6 kinases have been approved as therapeutic agents for estrogen receptor-positive breast cancers (Spring et al., 2020). While genomic data suggest an oncogenic role for CDK4/6 in EC, a Phase-II trial combining letrozole and abemaciclib in estrogen receptor–positive recurrent endometrial cancer reported a superior combination treatment efficacy (Konstantinopoulos et al., 2023). Collectively, all these observations suggested that CDK4/6 inhibitors might represent an attractive therapeutic strategy for EC. Given this background, CDK4/6 inhibitor combination regimens may be particularly attractive in EC.

To better comprehend the determinants for CDK4/6 inhibitor sensitivity in EC, we performed an unbiased genome-wide CRISPR-Cas9 knockout screen and identified NIMA-related kinase-6 (NEK6) as an essential determinant of EC cells vulnerability to CDK4/6 inhibitors. NEK6 is a serine/threonine kinase from the never in mitosis gene A (NIMA)-related kinase family involved in mitotic division (Sampson et al., 2017). NEK6 has been demonstrated to play a critical role in mitosis progression that includes mitotic spindle formation, metaphase to anaphase transition and centrosome separation (Fry et al., 2012). Interfering with NEK6 function could trigger cell cycle arrest and cell apoptosis. There is evidence that NEK6 is increased in several malignant human cancer cells including breast, colon, lung, kidney, rectum, thyroid, ovarian, prostate, pancreas and small intestine cancers, and correlates with patient clinical prognosis (He et al., 2018; Choudhury et al., 2017; Panchal and Evan Prince, 2023; Gao et al., 2025). Currently, the relationship between NEK6 and tumorigenesis has been preliminarily known, but its role in EC has not been reported yet.

Specifically, we revealed that the inhibition of NEK6 by genetic modification or ZINC05007751, a NEK6 selective inhibitor, could enhance the anti-tumor effects of palbociclib (a CDK4/6 inhibitor) in cell lines, patient-derived organoids and mouse models of EC. NEK6 can interact with YBX1 in the cytoplasm, and facilitate its phosphorylation at serine 102 (p-YBX1^S102^), which promotes YBX1 nuclear translocation and further activated CDK2 and bcl2 transcription. This cascade then induces DNA damage and cell apoptosis. Hence, combined inhibition of NEK6 and CDK4/6 represents a potential novel combination therapeutic strategy for EC patients.

## 2 Materials and methods

### 2.1 Cell culture and reagents

The human endometrial cancer cell line HEC-1A and ishikawa was purchased from the Cell Bank of the Chinese Academy of Sciences (Shanghai, China). The human 293T cell line was gifted from the Shanghai Institute of Endocrine and Metabolic Diseases. HEC-1A cells were cultured in McCoy’s 5A (Gibco, United States) supplemented with 10% fetal bovine serum (FBS). Ishikawa cells were cultured in Dulbecco’s Modified Eagle Medium (DMEM) medium (HyClone, United States) supplemented with 10% FBS. All of the above media contained 100 U/mL penicillin and 100 μg/mL streptomycin. All cells were cultured in a humidified incubator at 37 °C with 5% CO2. The CDK4/6 inhibitors (abemaciclib, palbociclib, ribociclib) and the specific NEK6 inhibitor ZINC05007751 was purchased from MedChem Express (Shanghai, China).

### 2.2 Western immunoblotting analysis

Western immunoblotting was performed as described previously (Jian et al., 2021). The following primary antibodies were used: anti-NEK6 antibody (1:1000, abcam, United States), anti-caspase-3 antibody (1:1000, Cell Signaling Technology, United States), anti-PARP antibody (1:1000, Cell Signaling Technology), anti-cleaved-caspase-3 antibody (1:1000, Cell Signaling Technology), anti-γH2AX antibody (1:1000, abcam), anti-flag antibody (1:1000, Cell Signaling Technology), anti-YBX1 antibody (1:1000, Cell Signaling Technology), anti YBX1 antibody (phospho-S102) (1:1000, Cell Signaling Technology) and anti-GAPDH antibody (1:1000, Cell Signaling Technology).

### 2.3 Plasmids and stable cell lines

pRSET YBX1 plasmid was purchased from addgene (#13035, Cambridge, MA, United States). The target sequences of siRNAs (BioTNT, China) were provided in Supplementary Table S1. The siRNA transfections were conducted with the lipofectamine 2000 (Thermofishier scientific, United States) according to the manufacturer’s instructions. The NEK6 sequence was cloned into the lentiviral vector pSLenti-U6-shRNA-CMV-EGFP-F2A-Puro-WPRE for stable overexpression in EC cells. The shRNA sequences against NEK6 (1, CCC​GGA​GAG​GAC​AGT​ATG​GAA; 2, GAA​CCA​CCC​AAA​TAT​CAT​CAA; 3, CGA​AGA​CAA​CGA​GCT​GAA​CAT) were cloned into PGLV2/H1 (OBiO, China). Stable cell lines were generated through transduction with packaged lentivirus. Transduced cells were selected for stably infected cells with puromycin (2 μg/mL).

qRT-PCR, cell apoptosis and comet assay were performed as described previously (Jian et al., 2021). Primer sequences for qRT-PCR are provided in Supplementary Table S1.

### 2.4 Cell proliferation assay

Cell proliferation was detected using the Cell Counting Kit-8 (CCK-8) assay. In brief, cells were seeded at the appropriate density in a 96-well plate and treated as described. Then, the CCK-8 assay was performed using following the manufacturer’s protocol (MedChemExpress, China), and then cell proliferation was quantified by detecting the optical density at 450 nm (OD450) on a microplate reader (Tecan, Infinite 200 PRO). IC50 were calculated with the functions provided in GraphPad Prism software version 7.0. The synergistic effect of CDK4/6 inhibitor combined with NEK6 inhibitor was assessed using a dose-response matrix. The package SynergyFinder in R software (version 4.3.0) was used to calculate and visualize synergy scores according to the synergy scoring models-Loewe Additivity models.

### 2.5 Co-immunoprecipitation and mass spectrometry (Co-IP-MS)

The PierceTM Classic Magnetic IP/Co-IP Kit (Thermo Fisher, United States) was used (according to the manufacturer’s instructions) to examine proteins coupled to NEK6. 293T cells stably expressing 3×Flag-tagged NEK6 were washed with ice-cold Phosphate Buffered Saline (PBS) and then lysed in IP lysis buffer containing 1×Protease Inhibitor Cocktail. One-tenth of the supernatant was saved as the input. To the remaining supernatant, 5 μg flag antibody-magnetic bead complex was added and then samples were incubated at 4 °C, overnight. Beads were washed five times with buffer containing 10 μg/mL RNase A before being eluted by 100 μL elution buffer. Protein samples (20 μL) were boiled in SDS buffer for Western blotting, and 80 μL protein samples were analyzed by label-free LC/MS. The gel was stained by Coomassie Brilliant Blue and FLAG-NEK6 bands were excised for mass spectrometry analysis to identify potential binding protein.

### 2.6 Immunohistochemistry (IHC)

*Cancer tissues and adjacent tissues of 90 EC samples* were prepared in a tissue microarray. Briefly, a hollow needle was used to acquire tissue cores as small as 1.5 mm in diameter from paraffin-embedded surgical specimens of EC patients. These tissue cores are then inserted in a recipient paraffin block in a precisely spaced, array pattern. Sections from this block are cut and mounted on microscope slides, and then analyzed IHC. Briefly, the sections were deparaffinized in xylene and rehydrated through a graded ethanol series and then subjected to antigen repair by citric acid with the microwave boiling method. Endogenous peroxidase activity was blocked by incubation with 3% hydrogen peroxide at room temperature for 10 min. Then, the slides were incubated with antibodies overnight at 4 °C. The next day, sections were washed in PBS three times for 5 min each time. Secondary antibody was applied for 30 min at 37 °C, and color was developed with a diaminobenzidine peroxidase substrate kit (Impact DAB, Vector Laboratories, United States). Sections were then counterstained with hematoxylin, dehydrated and mounted. Images were acquired with a thunder imager Leica DM6B and the confocal microscopy (Olympus, Japan). IHC staining was semi-quantitatively evaluated based on stain intensity and the percentage of positive cells. Staining intensities were categorized as 0 (negative), 1 (weakly positive), 2 (moderately positive) or 3 (strongly positive). The percentage of positive cells were categorized as 1 (0%–10%), 2 (11%–50%), 3 (51%–80%) or 4 (81%–100%). The final score for each section was determined by multiplying the staining intensity score by the percentage staining score.

### 2.7 Immunofluorescence

Staining of cultured cells were fixed for 10 min in 4% paraformaldehyde. The fixed cells were permeabilized with 0.3% Triton X-100 in PBS for 2 min on ice. Samples were then washed with PBS before blocking overnight with 2.5% bovine serum albumin (BSA). Blocked samples were then incubated overnight at 4 °C with primary antibodies against human NEK6 antibody (1:100, abcam, United States), YBX1 antibody (1:100, Cell Signaling Technology) or γH2AX antibody (1:500, abcam). Primary antibodies were removed and the samples were washed with PBS before staining with secondary antibodies at room temperature for 1 h. After washing three times with PBS, the slides were mounted with antifade reagent with DAPI.

The organoids were permeabilized with 0.5% Triton-X 100/PBS for 30 min. The blocking steps were performed with 10% goat serum. The primary antibody ki67 (Beyotime, Haimen, China) was added and incubated at 4 °C overnight with gentle shaking. After washing, the organoids were incubated with secondary antibody for 2 h. After washing, DAPI was added as a nuclear counterstain and the slides were mounted in ibidi Mounting Medium.

### 2.8 Genome‐wide CRISPR/Cas9 screen

**FIGURE 1:**
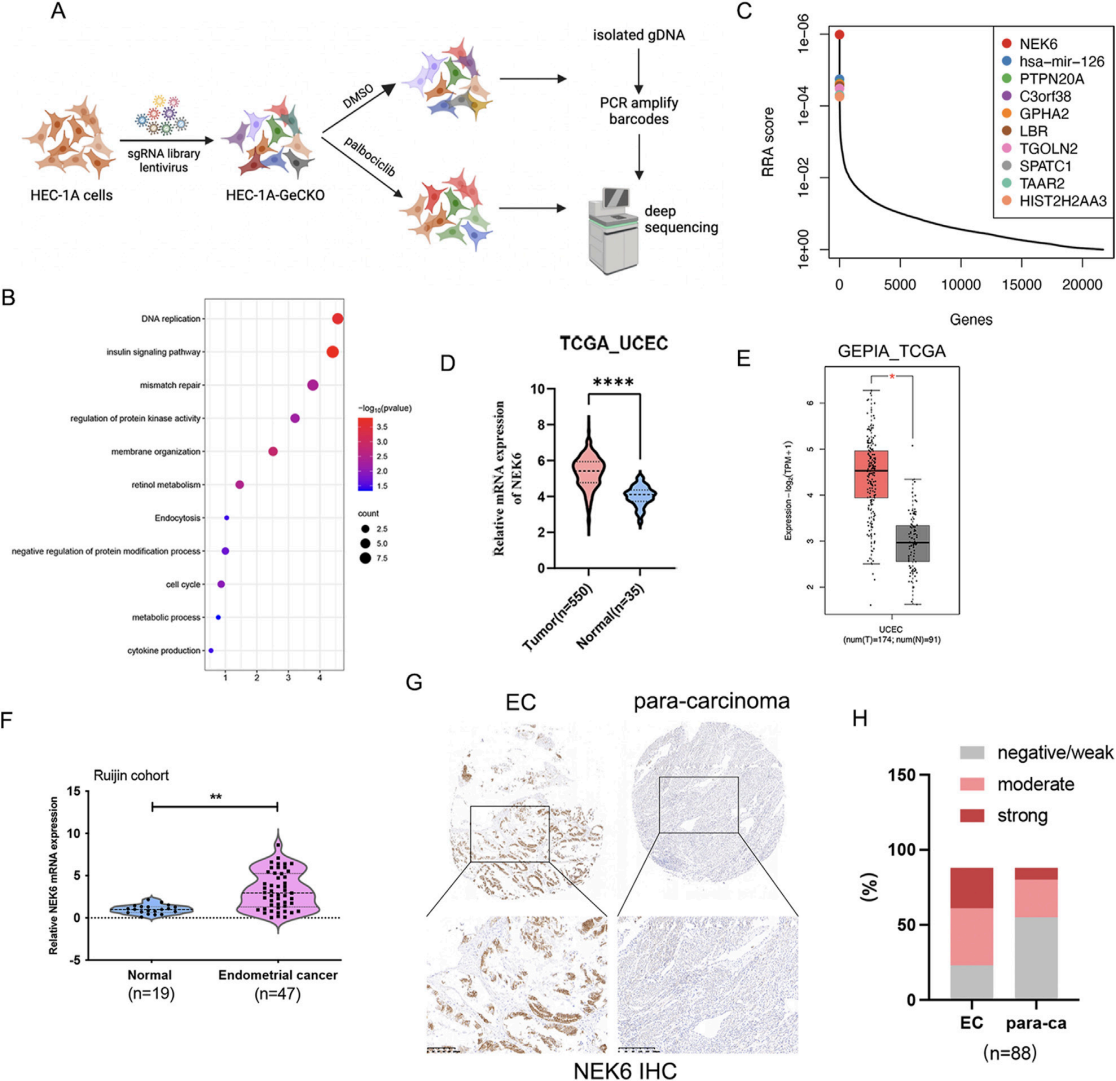
Genome-scale CRISPR knockout screen and candidate identification. **(A)** Schematic representation of the CRISPR knockout screen in human endometrial cancer HEC-1A cells. **(B)** KEGG analysis the top 250 palbociclib negatively selected hits in the CRISPR-Cas9 screen. **(C)** The top 10 candidates that were most significantly depleted after palbociclib selection are ranked by a modified robust ranking aggregation (RRA score). **(D)** NEK6 expression in endometrial cancer was analyzed using RNA-Seq datasets from the TCGA database. **(E)** NEK6 gene expression level from GEPIA (http://gepia.cancer-pku.cn). It includes 174 TCGA-UCSC samples and 91 GTEx normal endometrial samples. **(F)** NEK6 was detected in 47 EC tissues and 19 normal endometrial tissues by qRT-PCR. **(G)** Representative images of NEK6 in paracancerous and EC tissues detected on a TMA. **(H)** Quantification of NEK6 staining intensities in human EC. The Y-axis shows the percentage of tumors with negative/weak, moderate and strong IHC staining.

Human GeCKO v2 CRISPR knockout pooled library was a gift from Feng Zhang (Addgene # 1 000 000 048). Briefly, the human GeCKO v2 CRISPR library, containing 123,411 unique sgRNAs targeting 19,050 protein-coding genes, was used to generate a mutant EC cell pool by transduction of HEC-1A cells at a low multiplicity of infection (MOI = 0.3) followed by puromycin selection (Figure 1A; Supplementary Figure S1A). Expression of Cas9 in this stable cell line is confirmed by Western blot (Supplementary Figure S1B). The cell viability assay shows that Cas9 expression and puromycin selection has a minimal effect on sensitivity to palbociclib in HEC-1A-Cas9 cells, comparing to the parental HEC-1A cells (Supplementary Figure S1C). The stably transfected cells were treated with DMSO or palbociclib (IC30 2 μM) respectively for 3 days and then they were restored in normal medium for 14 days to enable negative screening. Then, genomic DNA from approximately 5 × 10^7^ cells were isolated by a Quick-DNA Midiprep Plus Kit (D4075, Zymo research, United States) and subjected to PCR to construct the sequencing library. The forward primer sequence for sgRNA library amplification and next-generation sequencing (NGS) was 5′-AAT​GAT​ACG​GCG​ACC​ACC​GAG​ATC​TA CACTCT TTC​CCT​ACA​CGA​CGC​TCT​TCC​GAT​CTA​TCA​TGC TTAGCTTTATATATC TTGTGGAAAGGACGAAAC ACC-3′. The reverse primer was 5′-CAAGCAGAAGAC GGC​ATA​CGA​GAT​GAA​GAA​GTG​TGA​CTG​GAG​TTC AGA​CGT​GTG​CTC​TTC​CGA​TCT​CCG​ACT​CGG​TGC CACTTTTTCAA-3′ for the control group and 5′-CAA GCA​GAA​GAC​GGC​ATA​CGA​GAT​AT TCTAGGGTG ACT​GGA​GTT​CAG​ACG​TGT​GCT​CTT​CCG​ATC​TCC GACTCGGTGCC ACTTTTTCAA-3′. sgRNA abundance was determined by high-throughput sequencing and analyzed by MAGeCKFlute. sgRNA abundance was determined by high-throughput sequencing and analyzed by MAGeCKFlute (Wang et al., 2019).

### 2.9 Xenograft mouse model

For the *in vivo* tumor formation assays, female BALB/c nude mice (four to five weeks old) (Shanghai Lingchang Biology Co., Ltd., China) were used to establish xenograft tumors derived from either HEC-1A-shNC or HEC-1A-shNEK6 cells. In all, 1 × 10^7^ were suspended in 200 μL of serum-free DMEM and subcutaneously injected into the flank of each nude mouse. When tumor volume reached 100 mm^3^ on average, HEC-1A-shNC or HEC-1A-shNEK6 transplantation nude mouse were randomized to treatment with vehicle or palbociclib treatment (50 mg/kg, daily) daily gavage. Palbociclib oral solution was prepared in 0.5% methylcellulose. The body weight, tumor sizes and mouse weights were measured twice weekly, and tumor volumes were calculated as V (mm^3^) = width^2^ (mm^2^) × length (mm)/2. After 21 days of treatment, all nude mice were sacrificed and tumors were collected. The tumor weight was recorded. Animal studies were performed in accordance with protocols approved by the Animal Ethics Review Committee of Shanghai-Jiaotong University School of Medicine.

### 2.10 Human EC organoids cultures and assays

Organoids were generated according to previously described protocols (Bredenoord et al., 2017; Boretto et al., 2019) with slight modifications. Briefly, primary human endometrial tumor tissues (0.25–1 cm^3^) were minced. After digesting with collagenase at 37 °C for 60 min with gentle shaking, leave the supernatant at room temperature for 3 min. The supernatant was filtered through one or two sieves (100 μm). The epithelial components were washed twice and collected by centrifugation and then mixed with ice-cold Matrigel for organoid establishment. Organoids were allowed to form for 3 weeks with Endometrial Cancer Organoid Medium (BioGenous, China) containing necessary cytokines and recombinant proteins refreshed every 3 days. Cell line-derived organoids were plated at a density of 2,000 cells per well in 96-well plates embedded in Matrigel as hanging drops (5 μL per well). Organoids were treated with ZINC05007751 (1 μM), palbociclib (5 μM) or both for 7 days. Cell viability was assessed using a 3D CellTiter-Glo assay (Promega, United States) by quantifying metabolically active cells releasing ATP. Patients’ information is provided in Supplementary Table S2.

### 2.11 Chromatin immunoprecipitation analysis

Chromatin immunoprecipitation was performed using a kit according to the manufacturer’s instructions (Cell Signaling Technology; #9003). Antibodies against YBX1 (20339-1-AP, proteintech, United States) was used for ChIP. ChIP DNA was used for ChIP-qPCR or ChIP-seq. Oligonucleotide primers used for ChIP-qPCR analysis are listed in Supplementary Table S1.

### 2.12 Luciferase reporter assays

HEC-1A cells were plated 1 day before transfection. Cells were transfected the next day with 0.5 μg YBX1 and 0.5 μg CDK2-Luc reporter gene (or bcl2-luc reporter gene) plus 0.25 ng Renilla luciferase internal normalization plasmid (phRL-CMV) for each well with Lipofectamine 2000. Cells were serum-starved 24 h after transfection and then cultured for 24 h before measuring. Relative firefly luciferase activity was normalized to Renilla luciferase activity.

### 2.13 Statistical analysis

Data were presented as the means ± S.D. Two tailed-unpaired Student’s t-test was applied to compare the difference between two groups, and one-way analysis of variance test was applied to compare the difference among three or more groups. For survival analysis, the Kaplan-Meier method and log-rank test were applied to determine the OS. Pearson correlation coefficient was used to analyze the correlation of histoscore in IHC staining. All statistical calculation was performed using SPSS software package (version 23.0, IBM SPSS). P < 0.05 was considered statistically significant.

### 2.14 Ethics statement

This study was approved by Ethical Committee of Shanghai Jiao-tong University school of medicine, and human EC tissues were obtained with written informed consent.

## 3 Results

### 3.1 A genome‐wide sgRNA library screen identified NEK6 as a modifier of palbociclib sensitivity in EC

To systematically identify the critical genes associated with CDK4/6 inhibitor sensitivity in EC, we performed a genome-wide CRISPR/Cas9 knockout screen in endometrial cancer. We obtained a subset of sgRNAs targeting 597 genes were significantly depleted (P < 0.05) in the palbociclib-treated cells when compared to vehicle control, indicating that these genes might be potential drivers for palbociclib resistance. Kyoto Encyclopedia of Genes and Genomes (KEGG) pathway enrichment analysis of the top 250 genes with the most significant *P* values showed an enrichment of genes involved in DNA replication, mismatch repair and insulin signaling pathway (Figure 1B). By comparison of control and palbociclib-treated groups, depleted gRNAs were identified using Model-based Analysis of Genome-wide CRISPR-Cas9 Knockout (MAGeCK) tool. This analysis gave a ranking list based on the RRA score. Among the list of genes, NEK6 was identified as the most negatively selected gene upon palbociclib treatment (Figure 1C). All NEK6 targeting sgRNAs were dramatically decreased in palbociclib-treated cells (Supplementary Figure S2A). In addition, several previously reported genes related to the sensitivity of CDK4/6 inhibitor were identified, including FAT1, CDK2 and WEE1 (Pandey et al., 2020; Li et al., 2018; Fallah et al., 2021), providing internal validation of this approach. Furthermore, we found that NEK6 mRNA levels were elevated in EC in TCGA database (Figures 1D,E). Consistent with results of TCGA database analysis, NEK6 mRNA was also upregulated in EC samples compared with normal samples in our cohort (Figure 1F). Next, we detected the protein level of NEK6 in tissue microarrays (TMA) composed of 88 paired EC tissues and adjacent normal tissues. The results showed that the proportion of increased NEK6 protein levels were obviously higher in EC tissues compared with that in matched para-cancerous tissues (65/88 vs. 33/88 [73.8% vs. 37.5%], respectively) (Figures 1G,H). The above results indicated that NEK6 may be a potential contributor to CDK4/6 inhibitor sensitivity in EC.

### 3.2 NEK6 depletion enhances the sensitivity of CDK4/6 inhibitor in endometrial cancer cell lines

**FIGURE 2:**
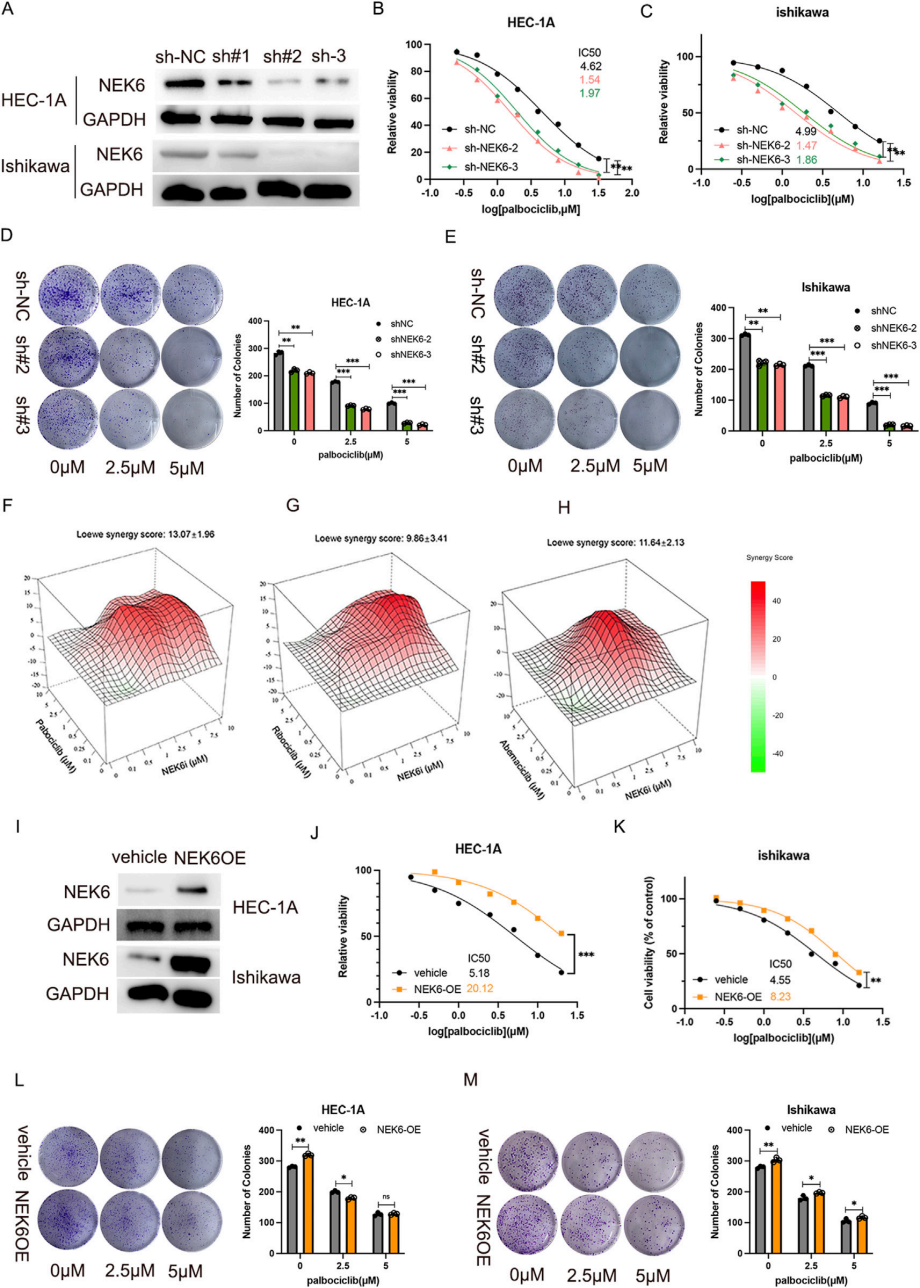
NEK6 knockout results in sensitivity to Palbociclib. **(A)** Western blot shows effective lentiviral shRNA-mediated knockdown of NEK6 in HEC-1-A cells. **(B)** The inhibition of NEK6 sensitizes EC HEC-1A cells to Palbociclib. **(C)** The inhibition of NEK6 sensitizes EC Ishikawa cells to Palbociclib. **(D,E)** Colony formation assay were performed in the indicated EC cell lines with or without Palbociclib treatment. F-H, Combenefit plots for combination-dose response of NEK6i with palbociclib **(F)**; NEK6i with abemaciclib **(G)** and NEK6i with ribociclib **(H)** in HEC-1A cells. Synergy mapped to drug-dose response using the LOEWE model. Higher positive scores indicate greater synergistic activity. Error bars = Mean ± SEM (n = 3). **(I)** NEK6 overexpression HEC-1A stable cell line was generated and validated by Western blot. **(J)** NEK6 overexpression decreased EC HEC-1A cells sensitivity to palbociclib. **(K)** NEK6 overexpression decreased EC ishikawa cells sensitivity to palbociclib. **(L,M)** Overexpression of NEK6 desensitizes HEC-1A and Ishikawa cells to palbociclib treatment in clonogenic assay. Vehicle cells and NEK6OE cells were exposed to increasing concentrations of palbocilib and grew for 10 days. Images of colonies in colony formation assay were presented on the left. The quantitative results of duplicate biological experiments are shown on the right.

To investigate the effects of NEK6 on CDK4/6 inhibitor resistance, we generated stable NEK6 knockdown subclones in HEC-1A and ishikawa cells by shRNA (Figure 2A). We found that the knockdown of NEK6 could increase the sensitivity of HEC-1A and Ishikawa cells to CDK4/6 inhibitors (Figures 2B,C). Clonogenic assays shown that combined inhibition of CDK4/6 and NEK6 significantly decreased the number of colonies when compared with inhibition of NEK6 or CDK4/6 alone (Figures 2D,E). Further, synergistic inhibitory effect of NEK6 inhibitor and three structurally distinct CDK4/6 inhibitors (abemaciclib, palbociclib and ribociclib) was evaluated. Using the Loewe Synergy Score, we found a synergistic interaction between CDK4/6 inhibitor and NEK6 inhibitor, with the strongest synergy score (13.07) observed in the palbociclib (Figures 2F–H). In contrast, stable ectopic expression of NEK6 in HEC-1A and ishikawa cell lines desensitized cancer cells to palbociclib, evidenced by an increased IC50 and clonogenic assays (Figures 2I–M). These results suggested that sensitivity of CDK4/6 inhibitor was correlated with the expression level of NEK6.

### 3.3 Inhibition of NEK6 sensitized EC cells to CDK4/6 inhibitor treatment in EC organoids and *in-vivo*

**FIGURE 3:**
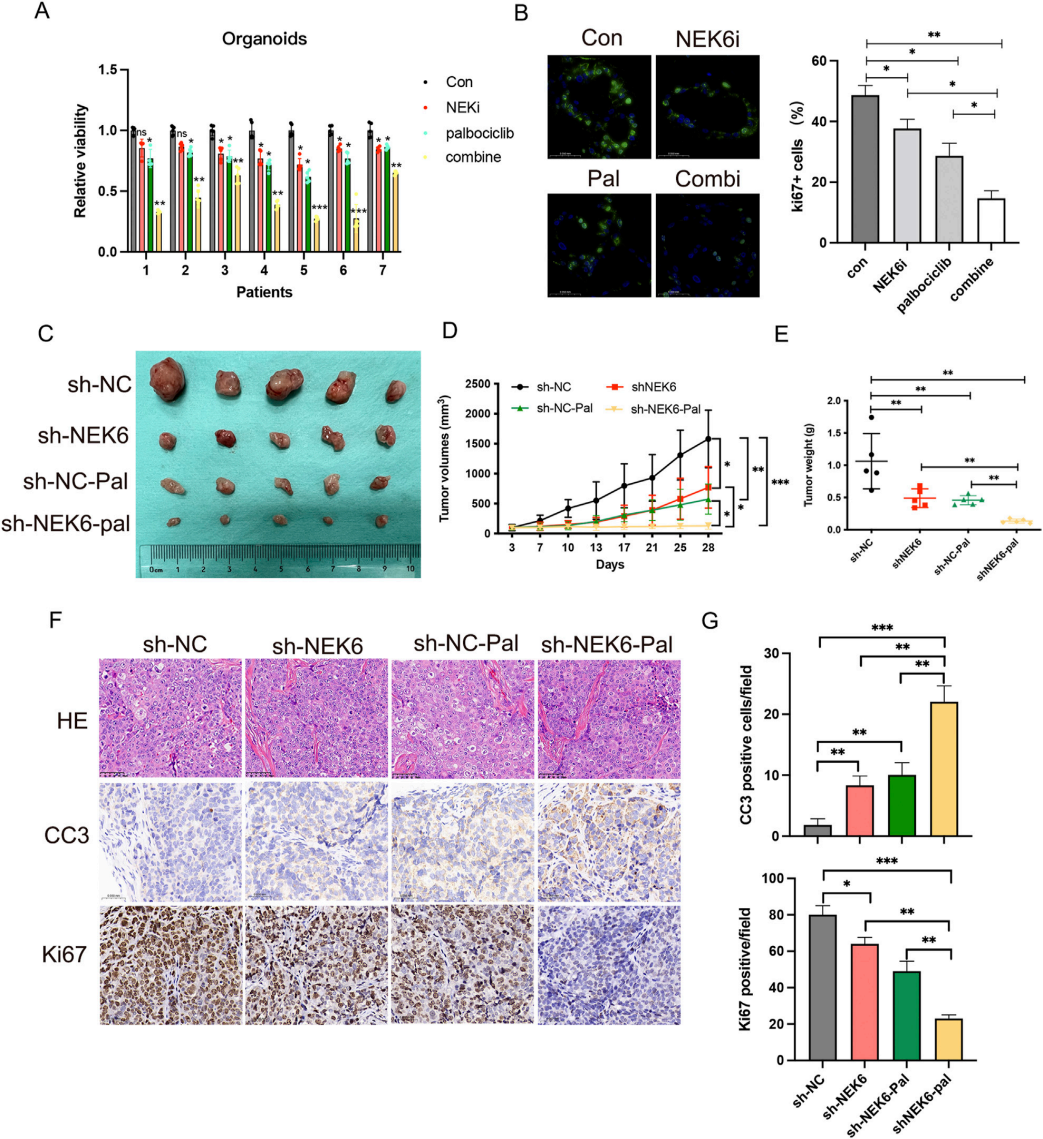
Inhibition of NEK6 sensitizes HEC-1A-derived xenograft tumors and organoids to palbociclib treatment. **(A)** Seven patients’ organoids (No.1-No.7) were respectively treated with DMSO, ZINC05007751 (1 μmol/L), palbociclib (5 μmol/L) or combined therapy for 6 days. Relative cell viability was analyzed. **(B)** Ki67 immunofluorescence of organoids treated with indicated inhibitors. The quantification of Ki67+ cells in organoids from three independent donors (right; bars show the mean of three technical replicates) and ki67 immunoflurescence (left; a representative image of three independent experiments). Scale bar, 50 μm. **(C)** HEC-1A-ShNC or HEC-1A-shNEK6 cells were subjected to xenograft assay and treated with palbociclib (50 mg/kg, by oral gavage) or drug vehicle. Mice were treated for 3 weeks and then killed. n = 5 for each group. **(C)** Xenograft tumors are shown. **(D)** Tumor growth curves of mice after the subcutaneous injection of HEC-1A cells expressing sh-NC or shNEK6 and intragastric administration of palbociclib, Data are presented as mean ± SD. **(E)** The weights of the tumors were quantified. *P < 0.05, **P < 0.01. **(F)** Immunostaining images of Ki67 and cleaved caspase-3 (CC3). Scale bar, 50 mm. **(G)** Quantification of data from **(F)**.

To further characterize the role of NEK6 in determining palbociclib sensitivity in pre-clinical models, we established patient-derived EC organoids for further analyses. Consistent with our observation in EC cell lines, combined treatment of NEK6 and CDK4/6 inhibitor could profoundly inhibit the proliferative potential of EC organoids, as reflected by CCK8 assay and the number of Ki67+ cells (Figures 3A,B). To explore whether these *in vitro* findings could be recapitulated *in vivo*, we employed a nude mouse model. Nude mice with established xenograft tumors derived from either HEC-1A-shNC or HEC-1A-shNEK6 cells were treated with either palbociclib or drug vehicle for 21 days. We found that HEC-1A-shNC-derived tumor growth could be suppressed by palbociclib, while loss of NEK6 significantly enhanced the growth inhibition (Figures 3C–E). Moreover, immunohistochemical staining of Ki67 and cleaved-caspase3 indicated that palbociclib could inhibit the proliferation and promote apoptosis of EC cells under NEK6 knockdown (Figures 3F,G). The body weight were comparable between these four groups (Supplementary Figure S3A). Together, these data demonstrate that NEK6 depletion enhanced the sensitivity of EC cells to CDK4/6 inhibitor both *in vivo* and *in vitro*.

### 3.4 Combined inhibition of CDK4/6 and NEK6 leads to increased DNA damage and cell apoptosis

**FIGURE 4:**
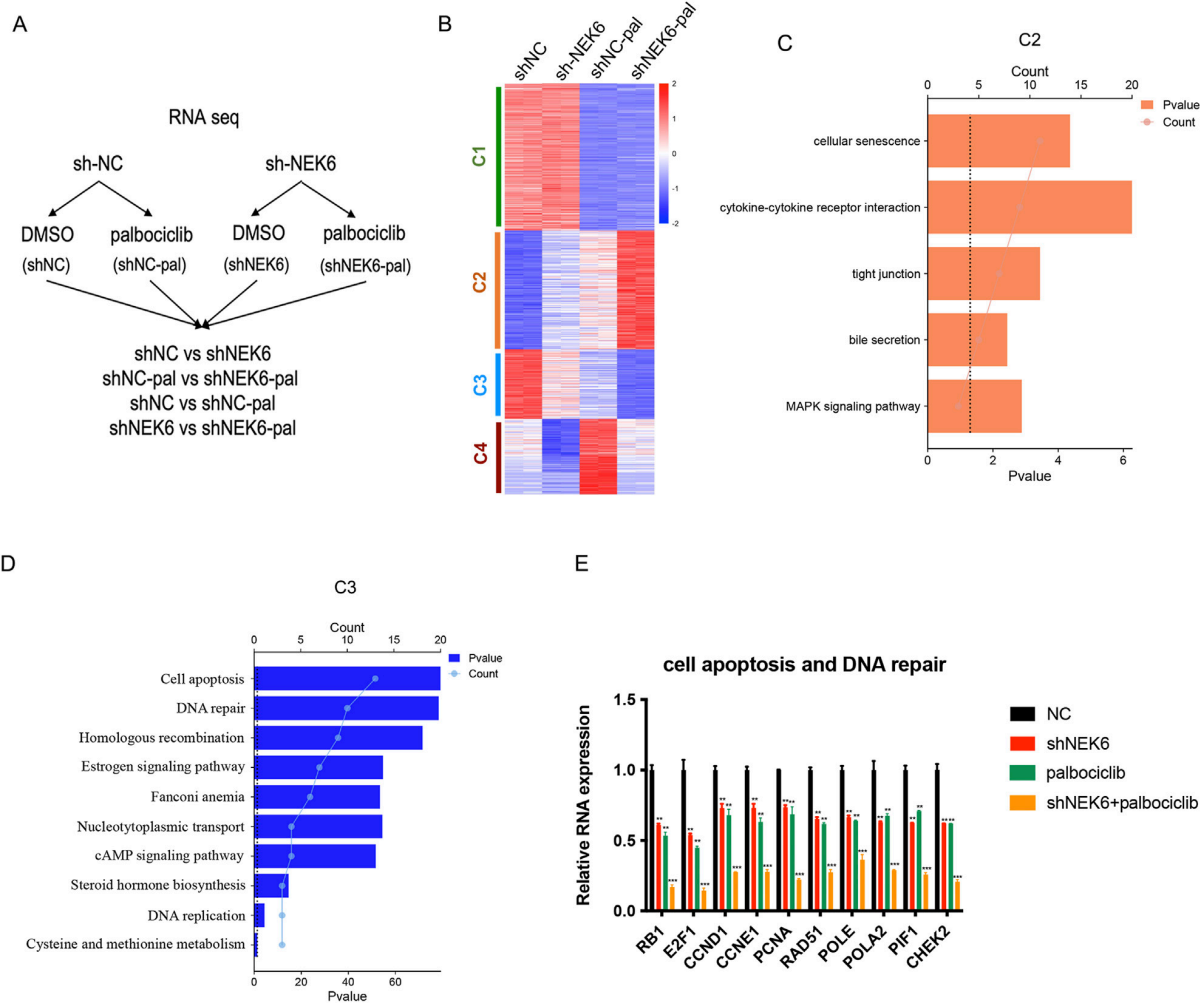
NEK6 modulates the expression of palbociclib-regulated genes. **(A)** Experimental design of RNA-seq experiment. HEC-1A shNC and HEC-1A shNEK6 cells were treated with Palbociclib or DMSO. **(B)** Heatmap of RNA-seq data in shNC, shNC-pal, shNEK6, and shNEK6-pal. Clusters were generated using K-means clustering function in Morpheus software. **(C,D)** Top significant pathways enriched in C2 and C3, respectively. **(E)** qRT-PCR validation of representative genes involved in the cell apoptosis and DNA repair. Data shown represent the mean ± s.d. of biologic triplicates.

To study the molecular mechanism underlying the antitumor effect of combination therapy, we examined global transcriptional changes by RNA-sequencing using HEC-1A-shNC or HEC-1A-shNEK6 cells that are treated with either vehicle or palbociclib (Figure 4A). We compared differentially regulated genes which subdivided into 4 major clusters by unsupervised clustering (Figure 4B). Cluster 2 included 127 genes that are synergistically induced in NEK6 knockdown + palbociclib group and these genes were mainly enriched in regulation of cellular senescence, cytokine-cytokine receptor interaction, tight junction, bile secretion and MAPK signaling pathway (Figure 4C). Cluster 3 included 67 genes that are synergistically repressed in NEK6 knockdown + palbociclib group and these genes were enriched in cell apoptosis, DNA repair, cell adhesion, homologous recombination, estrogen signaling pathway (Figure 4D). To verify the RNA-Seq results, 10 genes involved in regulation of cell apoptosis and DNA repair (RB1, E2F1, CCND1, CCNE1, PCNA, RAD51, POLE, POLA2, PIF1 and CHEK2) were selected and analyzed with qRT-PCR (Figure 4E). Results showed these genes decreased upon palbociclib, while knocking down NEK6 could further inhibit their expression. These data are consistent with data obtained by RNA-Seq analysis.

### 3.5 NEK6 knockdown enhances DNA damage and cytotoxicity of CDK4/6 inhibitor

**FIGURE 5:**
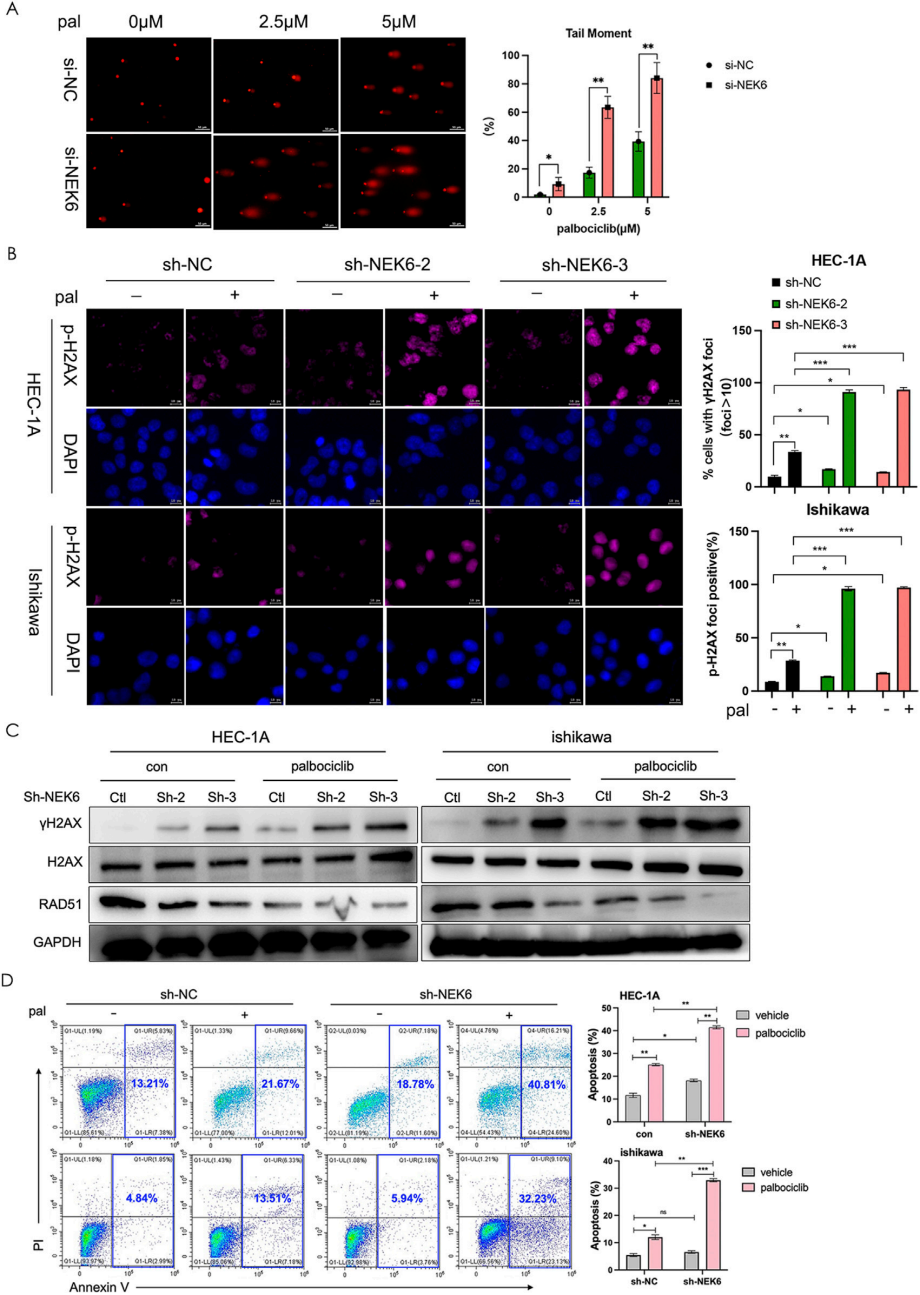
NEK6 knockout enhanced the efficiency of CDK4/6 inhibitor-induced DNA damage repair and apoptosis. **(A)** DNA damage detected by comet assay in HEC-1A cells (siNC or si-NEK6) treated with indicated concentration of palbociclib. Scale bar, 50 μm. Quantification of DNA in the tail from three independent experiments is shown as mean ± s.d. **(B)** Immunofluorescence staining of γ-H2AX in sh-NC and sh-NEK6 HEC-1A and Ishikawa cells treated with or without palbociclib. Scale bar = 10 μm. **(C)** Western blot analysis of phosphorylated H2AX and RAD51 in HEC-1A and ishikawa endometrial cancer cells treated as indicated. **(D)** Knockdown of NEK6 induced apoptosis significantly upon palbociclib treatment in HEC-1A evaluated by flow cytometry analysis. Data were presented as the mean ± s.d. of values obtained in 3 independent experiments. *, P < 0.05; **, P < 0.01; ***, P < 0.001; ns, not significant.

To confirm whether NEK6 inhibitors induce DNA damage in cancer cells by inhibiting DNA repair, we tested the effect of NEK6 inhibitor on palbociclib-mediated induction of DNA double-strand breaks (DSBs). DNA DSBs constitute a severe form of DNA damage that can be detected with the neutral comet assay. Neutral comet assays in HEC-1A cell lines showed inhibition of NEK6 or palbociclib modestly increased the percentage of tailed DNA, whereas combined inhibition of NEK6 and CDK4/6 significantly increased the percentage of tailed DNA (Figure 5A). Consistent with these findings, the levels of γ-H2AX (a marker of DSB damage and repair) of HEC-1A cells and Ishikawa cells were augmented by these combination treatments (Figure 5B). Subsequently, Western blotting assay revealed that NEK6 knockdown displayed higher levels of γH2AX and decreased RAD51 following palbociclib treatment (Figure 5C). Given that unrepaired DSB can trigger apoptosis, we further found that CDK4/6 inhibitor-induced apoptosis significantly increased in NEK6-knockdown cell lines (Figure 5D). Together, these results suggest that preventing DNA DSBs repair by NEK6 inhibition enhances the vulnerability of cancer cells to CDK4/6 inhibition.

### 3.6 Immunoprecipitation followed by mass spectrometry (IP‐MS) identified that NEK6 interacts with YBX1

**FIGURE 6:**
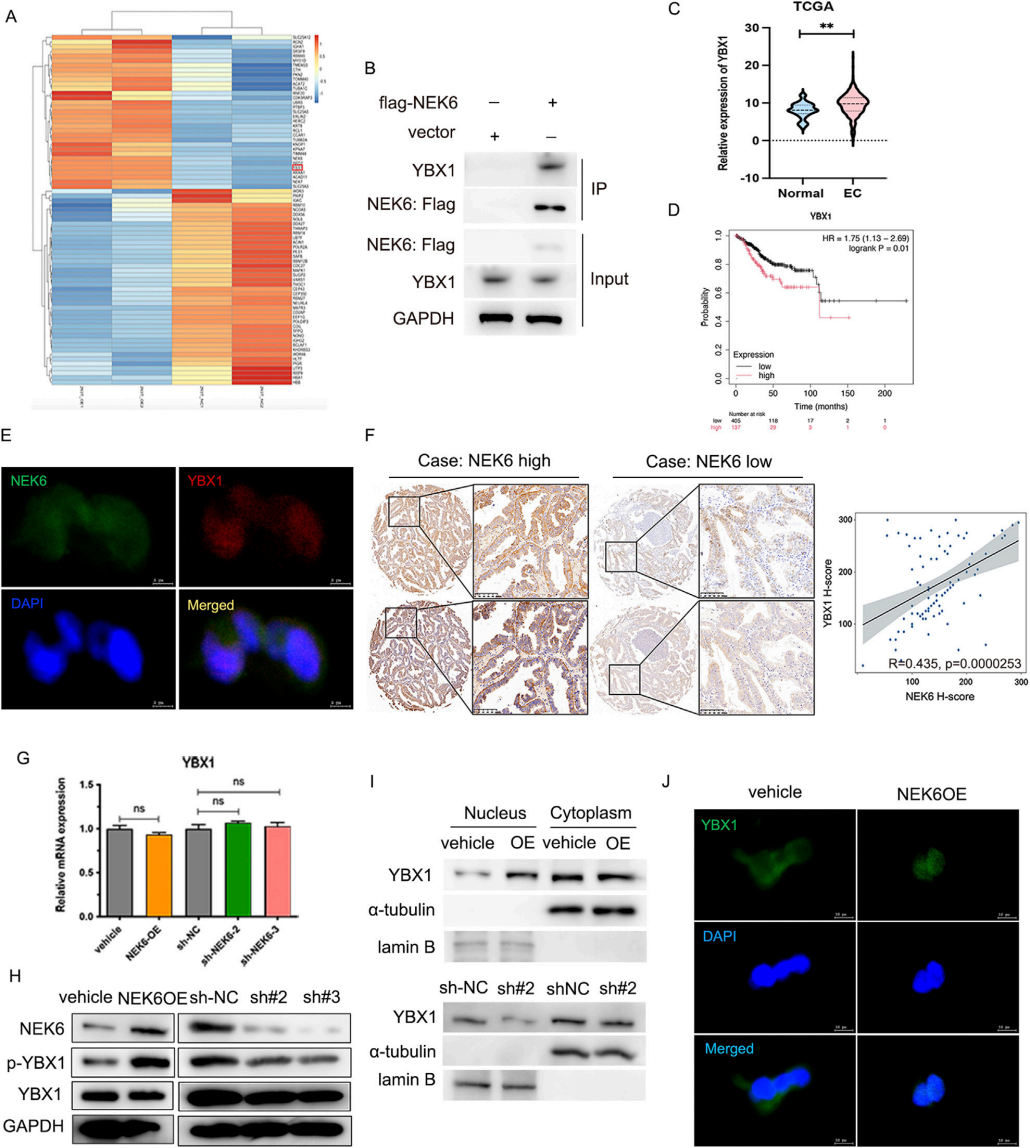
IP-MS indicates that YBX1 as a direct target of NEK6. **(A)** The heatmap of identified interacting proteins. **(B)** IP-western blot analysis validated the interaction of NEK6 and YBX1. **(C)** The YBX1 mRNA expression in endometrial cancer and normal breast tissues was analyzed based on TCGA databases. **(D)** the overall survival curve analysis of YBX1 between high and low YBX1 expression group in TCGA EC cohort. **(E)** The co-localization of NEK6 and YBX1 was assessed by immunofluorescence. Scale bar: 5 μm. **(F)** Representative immunohistochemical staining of NEK6 and YBX1 in the EC specimens on a TMA (n = 88). Linear regression analysis of the expression of NEK6 and YBX1. **(G)** YBX1 mRNA was determined by qRT-PCR after NEK6 overexpression knockdown in EC cells. **(H)** Altered nuclear translocation of YBX1 in response to NEK6 overexpression or knockdown in Ishikawa and HEC-1A cells analyzed by Western blotting. **(I)** The expression of YBX1 in nucleus and cytoplasm of NEK6-overexpressing HEC-1A cells were shown by Western blot. **(J)** immunofluorescence assay was performed to observe the localization of YBX1 in HEC-1A cells.

To gain insight into the mechanism by which NEK6 promotes the resistance of CDK4/6 inhibitor in EC, we conducted a systematic mass spectrometry analysis to identify NEK6-interacting proteins. 293T cells were transfected with adenovirus expressing Flag-tagged NEK6 or empty vector. Protein lysates were immunoprecipitated with anti-Flag M2–agarose. The immunoprecipitates were then eluted with Flag peptide, resolved by SDS-PAGE, detected by Coomassie blue staining, and identified by liquid chromatography–tandem mass spectrometry (LC-MS/MS). Based on this analysis, Y-box-binding protein 1 (YBX1) was found to be interacting with NEK6 (Figure 6A). YBX1 is a well-known oncogene that has been implicated in various types of cancers because it affects a wide range of genes involved in cell proliferation, survival, drug resistance and chromatin destabilization (Bergmann et al., 2005). Consistent with the IP-MS results, NEK6 pulled down YBX1 in HEC-1A cells, indicating that they interact (Figure 6B). Additionally, using bioinformatics data downloaded from TCGA EC databases, we detected the expression of YBX1 and prognosis in EC patients. The result showed that YBX1 was significantly increased in human EC tissues compared with normal endometrial tissues and was associated with unfavorable prognosis (Figures 6C,D). To further verify this result, immunofluorescence was used to observe the co-localization status of NEK6 and YBX1 (Figure 6E). We further evaluated the expression of YBX1 protein in cancerous and matched para-cancerous tissues in EC patients by IHC assays. As shown in Figure 6F, an increased YBX1 protein levels were obviously higher in EC tissues compared with that in matched para-cancerous tissues. The correlation analysis of NEK6 and YBX1 protein expression level was done, which showed that there was a positive correlation between NEK6 and YBX1 (P = 2.53E−5, r = 0.435) (Figure 6F). To explore the mechanism underlying the association between NEK6 and YBX1, we tested whether NEK6 affected YBX1 expression and found that NEK6 had no significant effect on YBX1 mRNA levels (Figure 6G). However, the YBX1 phosphorylation levels were dramatically increased under NEK6 overexpression, and were reduced under NEK6 silencing. (Figure 6H).

YBX1 is a nucleocytoplasmic shuttling protein and phosphorylation of YBX1 at ser102 induces its translocation to the nucleus. Furthermore, we overexpressed NEK6 in HEC-1A cells and found increased YBX1 levels in the nucleus concomitant with decreased YBX1 levels in the cytosolic fraction (Figure 6I). Likewise, the immunofluorescence results demonstrated that NEK6 overexpression promoted YBX1 nuclear translocation (Figure 6J).

### 3.7 YBX1 regulates the CDK2 and bcl2

**FIGURE 7:**
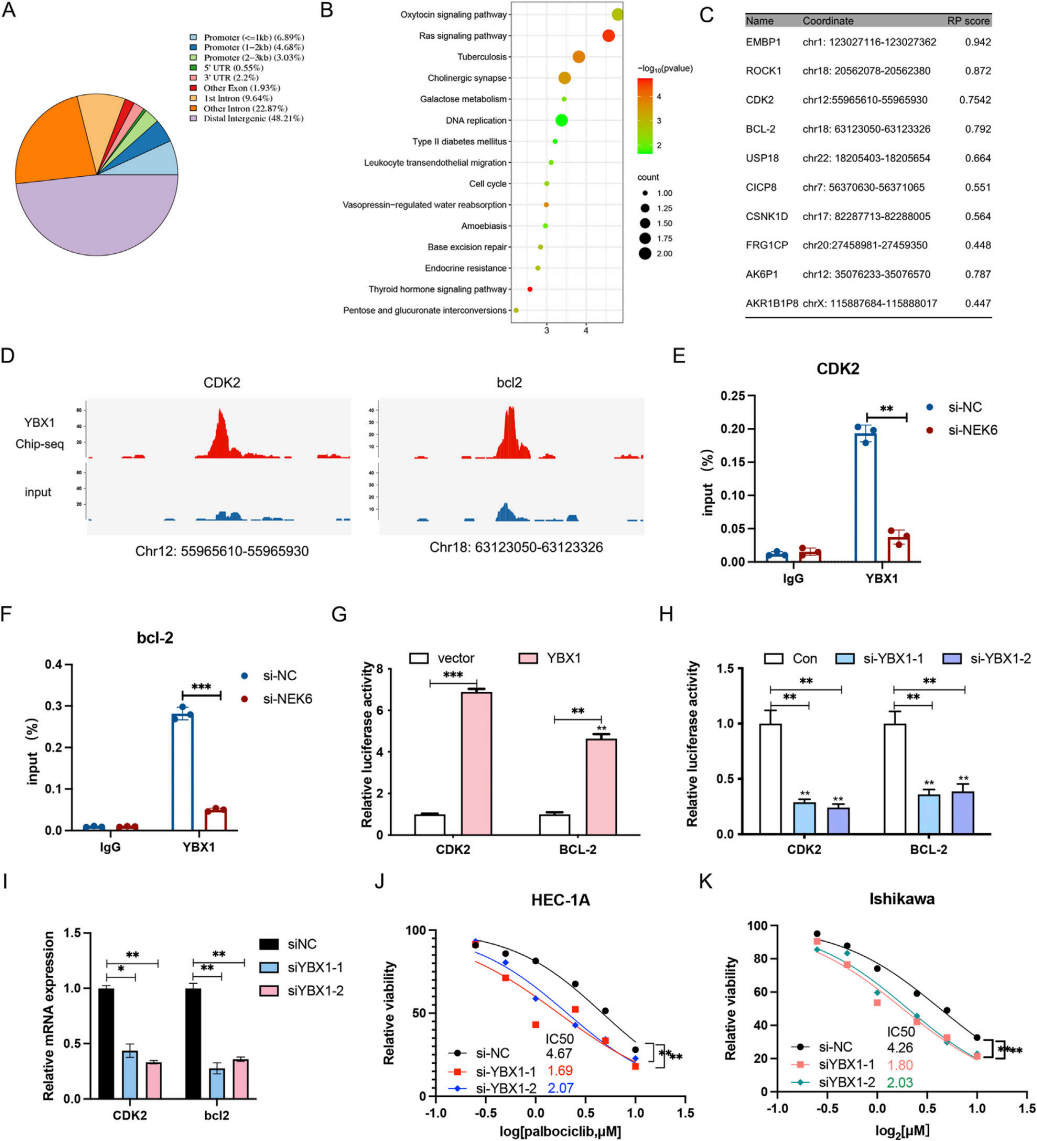
YBX1 regulates the transcription of CDK2 and bcl2. **(A)** The chart describes region types of YBX1 binding sites in genome. **(B)** KEGG analyses of the significant genes identified via ChIP-seq. **(C)** The top 10 target genes most likely regulated by YBX1. **(D)** Genome browser representation of YBX1 peaks at the promoters of CDK2 and bcl2. **(E,F)** ChIP-qPCR analysis showed that YBX1 specifically bound to the promoter region of CDK2 and bcl2, and NEK6 knockdown could decreased the binding of YBX1 to the promoter region of CDK2 and bcl2. **(G)** Luciferase reporter assay in 293T cells when YBX1 was overexpressed. **(H)** Luciferase reporter assay in 293T cells when YBX1 was knocked down. **(I)** the CDK2 and bcl2 mRNA level was determined by qRT-PCR after YBX1 knockdown in HEC-1A cells. **(J)** The inhibition of YBX1 sensitizes HEC-1A cells to palbociclib. **(K)** The inhibition of YBX1 sensitizes Ishikawa cells to palbociclib. *, P < 0.05; **, P < 0.01; ***P < 0.001; ns, not significant.

We then performed chromatin Immunoprecipitation followed by sequencing (ChIP-Seq) analysis for YBX1 in HEC-1A cells to identify critical and direct YBX1 targets. A total of 1,131 significant peaks and 524 peak-related genes were obtained, of which 14.6% were in promoters, 48.21% in intergenic regions distal to transcriptional start sites, and the remainder other introns, exons, 5′ UTR and 3′UTR of gene bodies (Figure 7A). KEGG pathway enrichment analysis of target genes indicated that they are involved in oxytocin signaling pathway, ras signaling pathway, DNA replication and cell cycle (Figure 7B). In addition, the ten most credible genes directly regulated by YBX1 were identified (Figure 7C). ChIP-seq showed YBX1 could bind to the CDK2 and bcl2 promoter (Figure 7D), which was validated by ChIP-qPCR (Figures 7E,F). To verify the influence of YBX1 on CDK2 and bcl2 transcription, we measured the luciferase activities of CDK2 and bcl2 promoter vectors in the presence of YBX1 plasmid or YBX1 siRNA. Luciferase reporter assays indicated that the luciferase activity for CDK2 and bcl2 promoter was dramatically enhanced by YBX1 co-transfected into EC cells (Figure 7G), while the luciferase activity and mRNA expression was significantly inhibited by YBX1-specific siRNA transfection (Figures 7H–I). We also found silencing YBX1 can significantly enhance the antiproliferative effect of palbociclib in EC cell lines (Figures 7J,K). Collectively, these results further confirm that the dysregulated NEK6/YBX1 axis is highly associated with palbociclib therapy resistance in EC patients, and its expression status might serve as a valuable biomarker to predict the combination therapy of CDK4/6 and NEK6 inhibitors sensitivity and survival outcomes of EC patients.

## 4 Discussion

Inhibitors of CDK4/6 kinases have been approved as a therapeutic agent for estrogen receptor-positive breast cancers, and they remarkedly improved the prognosis of a subset of patients (O'Leary et al., 2016; Iwata, 2018; Goel et al., 2022). We and others have observed anti-tumor efficacy of CDK4/6 inhibitors in EC preclinical models (Dosil et al., 2017). Given that CDK4/6 inhibitor monotherapy has not revealed evidence of remarkable efficacy to date in clinical studies, combination therapy will be critical. Some clinical trials are ongoing to determine the therapeutic benefit of palbociclib in combination therapy (with letrozole, gedatolisib or fulvestrant) for EC (NCT02730429, NCT03065062, NCT03643510). It is worth noting that palbociclib is the first CDK4/6-selective cycle inhibitor to demonstrate broad-ranging efficacy in many tumor types. Therefore, it seems reasonable to consider that palbociclib combinations potentially offer great promise for EC treatment.

To the best of our knowledge, our study is the first to perform an unbiased genome-wide CRISPR-Cas9 knockdown screen in an EC cell line to identify modifiers of CDK4/6 response. In the present study, we focused on the NEK6 gene (Panchal et al., 2023). NEK6 is frequently overexpressed in various cancerous tissues. It has a mechanistic role in DNA repair and could cause apoptosis when it is inhibited (Panchal et al., 2023). Gao found NEK6 was highly expressed in ovarian cancer (OC) and NEK6 knockdown led to inhibited growth, migration and invasion while promoting the apoptosis of OC cells (Gao et al., 2025). NEK6 was elevated in about 70% of hepatocellular carcinoma (HCC) cases and the expression of NEK6 was elevated in correlation with the progression of HCC tissue grade (Su et al., 2025). These findings indicate that NEK6 is a potential oncogene and an executable target in cancer. But its role in EC has not been established. Our study found that NEK6 was upregulated in human EC tissues, especially in endometrioid adenocarcinoma, the most common type of endometrial carcinoma (data not shown). We used 3 approaches (siRNA, lentivirus shRNA and a pharmacological inhibitor) to block NEK6 function and then examined the effects. The results showed that combination inhibition of NEK6 and CDK4/6 resulted in a stronger growth inhibition than either single inhibition, in EC cell lines, organoids and xenograft models. We found that NEK6 colocalized with YBX1 and could directly interact with YBX1. We demonstrated that the YBX1 is overexpressed in EC specimens and its overexpression correlated with poor prognosis, supporting an oncogenic role of YBX1 in cancer. Interestingly, we found that NEK6 may regulate the nuclear translocation of YBX1 by facilitating its phosphorylation at serine 102 (p-YBX1^S102^).

YBX1 is a member of the cold-shock domain protein family and is involved in several biological processes including transcription and translation regulation, mRNA precursor shearing, DNA repair and drug resistance (Lasham et al., 2013). YBX1 is primarily distributed in the cytoplasm. However, YBX1 can be phosphorylated at several critical serine residues resulting in YBX1 translocation into the nucleus. We found that NEK6 overexpression could increase YBX1 phosphorylation and promote the accumulation and nuclear translocation of YBX1 protein. Furthermore, we found that YBX1 bound to the CDK2 and bcl2 promoter and regulated these gene transcription, subsequently affecting cell cycle and apoptosis. CDK4/6 and CDK2 are key driving factors in the cell cycle process, and their functions are both sequential, partially overlapping, and cooperative, ensuring the correct progress of cell division. Interestingly, other groups have demonstrated that CDK2 and CDK4/6 inhibition arrests cells in G1 phase. Pandey et al. reported that the combined targeting of CDK2 and CDK4/6 synergistically inhibited cell proliferation (Pandey et al., 2020). These studies suggest that CDK2 and CDK4/6 co-inhibition as a therapeutic strategy in patients with advanced GIST (Schaefer et al., 2022). Interestingly, James et al. showed that combining Bcl2 and CDK4/6 inhibition elicits potent activity in endocrine-sensitive and refractory models of ER + breast cancer (Whittle et al., 2020). The dual inhibition of CDK4/6 with abemaciclib and Bcl-2 with venetoclax has been shown to induce synergistic anti-MCL activity in a variety of *in vitro* and *in vivo* preclinical relapsed/refractory MCL models (Che et al., 2023). Therefore, NEK6 regulates CDK2 and Bcl-2 through YBX1, which synergizes with CDK4/6 inhibitors to inhibit tumor growth. We then explored the co-regulated pathway by RNA-sequencing using HEC-1A-shNC or HEC-1A-shNEK6 cells treated with or without palcibiclib. Pathway analysis showed that the significant terms associated with the downregulated genes is cell apoptosis, DNA repair and spliceosome. We further found that inhibition of CDK4/6 impeded DNA DSB repair, induced DNA damage and cell apoptosis in NEK6-depleted cells.

**FIGURE 8:**
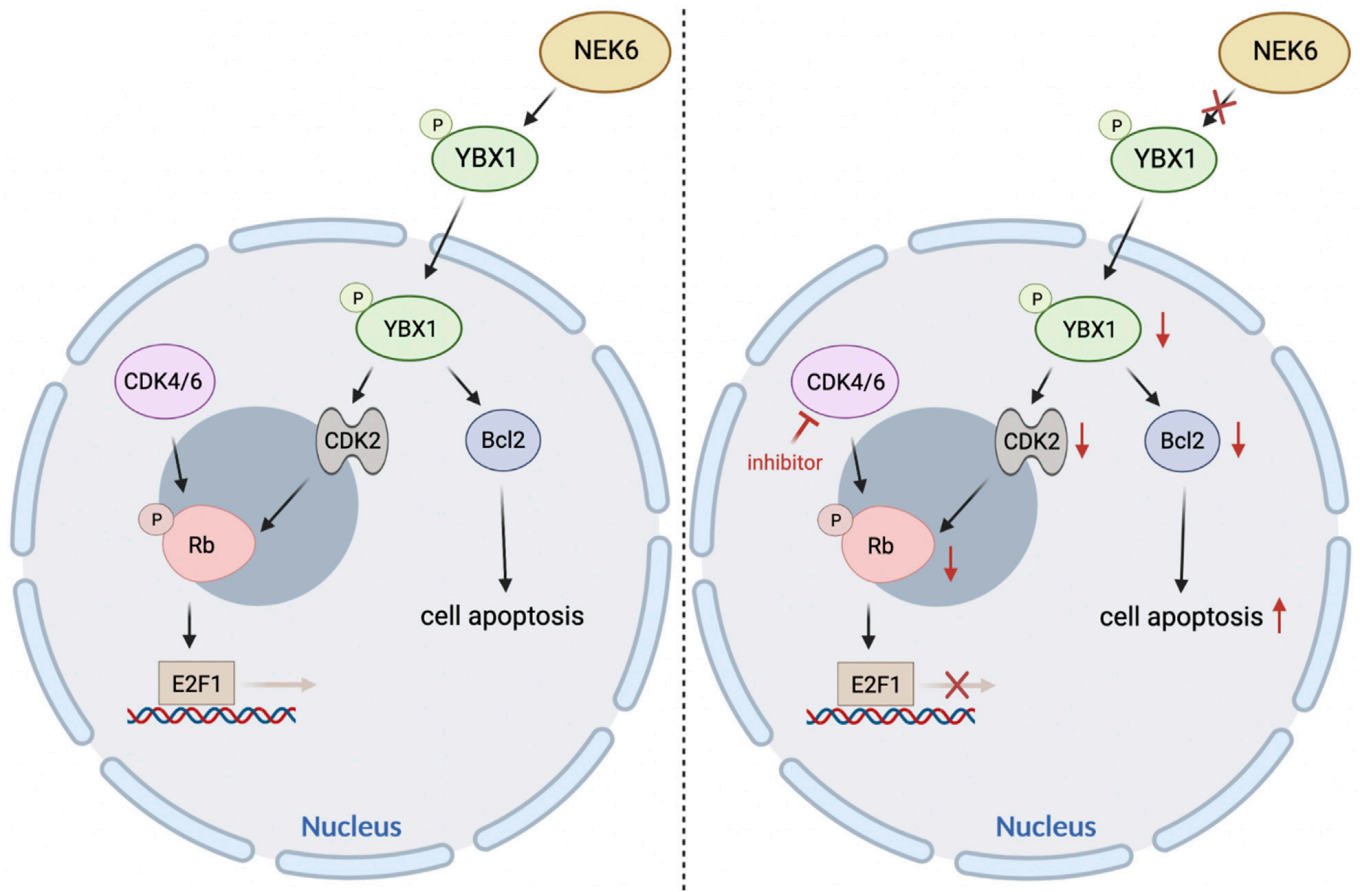
The proposed model elaborating the mechanisms underlying NEK6 depletion enhanced CDK4/6 inhibitors sensitivity in EC.

Taken together, these preclinical models demonstrated that cotargeting of NEK6 and CDK4/6 could be a viable strategy for the treatment of EC (Figure 8). Despite the promising responses observed in preclinical models of EC, it remains to be determined if the combination will be effective in the clinic.

## Data Availability

The data presented in the study are deposited in the NCBI SRA repository, accession number PRJNA1381769.
