# Supplementary material for: Genome-wide CRISPR screen identified NEK6 as a determinant of sensitivity to CDK4/6 inhibitor in endometrial cancer
Source: Front Pharmacol. 2026 Jan 5;16:1725886. doi: 10.3389/fphar.2025.1725886 (PMC12813190; doi:10.3389/fphar.2025.1725886)
Supplement: Supplementary file 1 [file Supplementaryfile1.docx]

**Supplementary Materials**

**Supplement Figures**

**
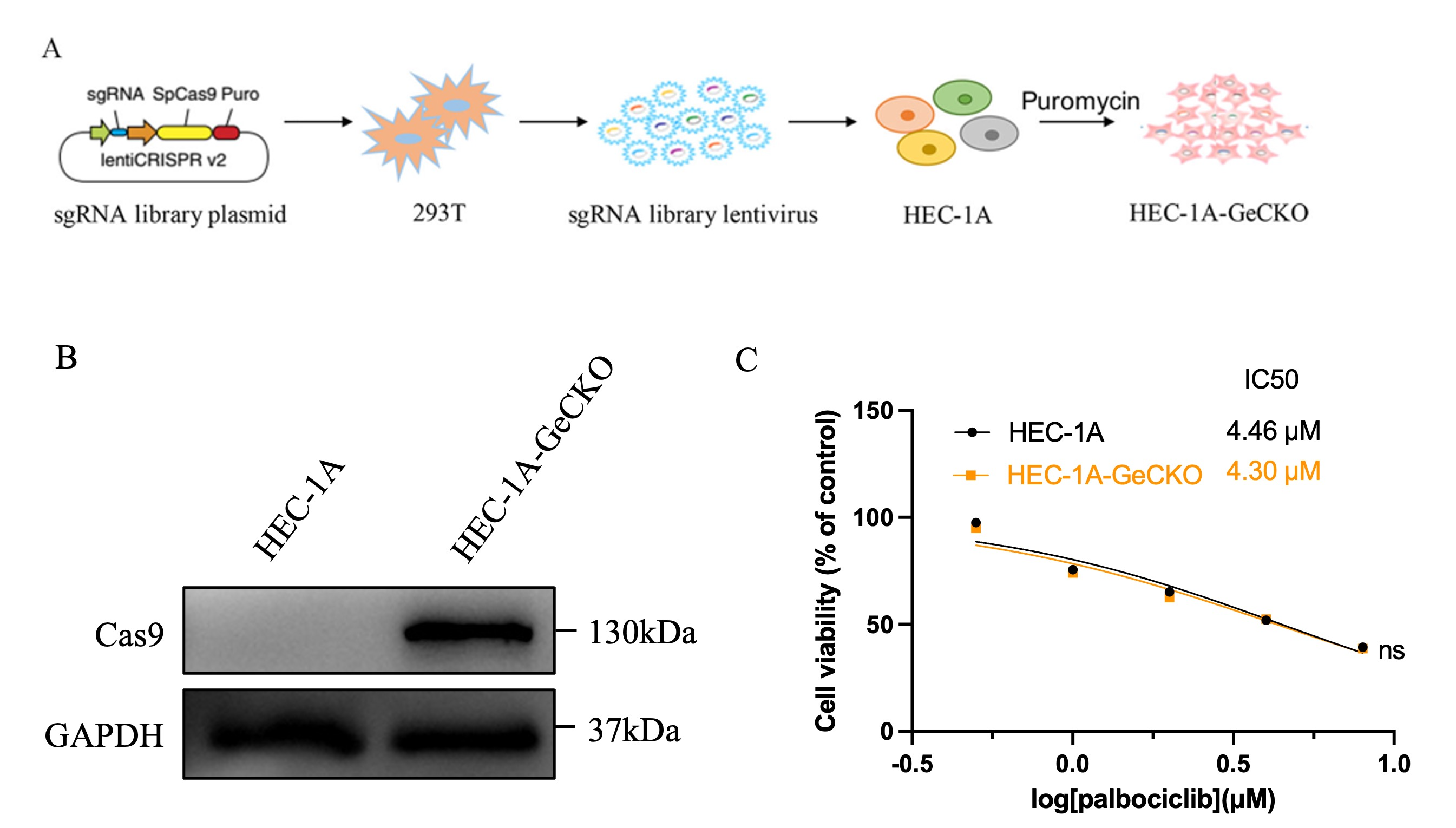
**

**Fig. S1** A, Schematic diagram illustrates the workflow of establishing HEC-1A-GeCKO cell line. B, Stable expression of Cas9 endonuclease in HEC-1A-GeCKO cell cells. FLAG-tagged Cas9 was detected by western blot with anti-FLAG antibody. C, HEC-1A-GeCKO and control cells exhibit similar viability under palbociclib treatment at indicated doses. IC50, 50% inhibitory concentration.

**
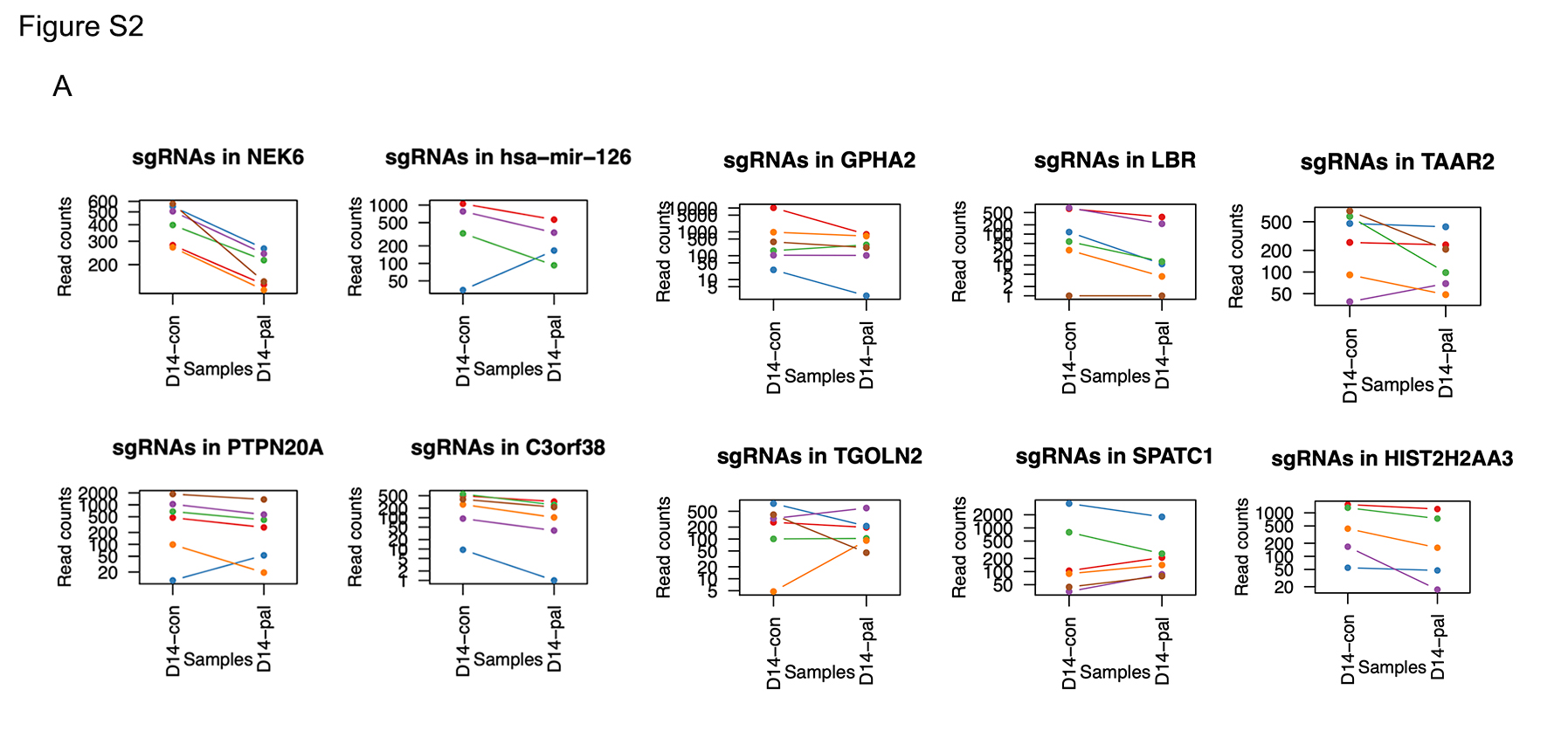
**

**Fig. S2 A.** sgRNA read counts of some selected gene which may be potentially critical for the maintenance of palbociclib resistance

**Fig. S3 A** Body weights of mice after the subcutaneous injection of HEC-1A cells expressing sh-NC or shNEK6 and intragastric administration of palbociclib, Data are presented as mean ± SD.

| Table S1:The list of primers and siRNA sequence. | | | |
| --- | --- | --- | --- |
| human qRT-PCR | Forward primer | | Reverse primer |
| YBX1 | | GGACAAGAAGGTCATCGCAAC | TCTCCATCTCCTACACTGCGA |
| GAPDH | | AGCCACATCGCTCAGACAC | GCCCAATACGACCAAATCC |
| RB1 | | TTGGATCACAGCGATACAAACTT | AGCGCACGCCAATAAAGACAT |
| E2F1 | | ACGCTATGAGACCTCACTGAA | TCCTGGGTCAACCCCTCAAG |
| CCND1 | | CAATGACCCCGCACGATTTC | CATGGAGGGCGGATTGGAA |
| CCNE1 | | ACTCAACGTGCAAGCCTCG | GCTCAAGAAAGTGCTGATCCC |
| PCNA | | ACACTAAGGGCCGAAGATAACG | ACAGCATCTCCAATATGGCTGA |
| RAD51 | | CAACCCATTTCACGGTTAGAGC | TTCTTTGGCGCATAGGCAACA |
| POLE | | TTGCGACCAGAAAGGGTTGT | TGATTTGGCAAGTCCAGATCCT |
| POLA2 | | GAAGCGAGCTATCTCTACCCC | CCACTTCTCCTCGGTTACTTCG |
| PIF1 | | CATCCACAAGAGCCAAGGCAT | GGTGGCATAGAAGTGCAGCA |
| CHEK2 | | TCTCGGGAGTCGGATGTTGAG | CCTGAGTGGACACTGTCTCTAA |
| CDK2 | | CCAGGAGTTACTTCTATGCCTGA | TTCATCCAGGGGAGGTACAAC |
| Bcl2 | | GGTGGGGTCATGTGTGTGG | CGGTTCAGGTACTCAGTCATCC |
| ChIP primers | | LEFT PRIMER | RIGHT PRIMER |
| CDK2 | | ATTCTGCTGGGGGAGGATCT | TCCTCTCCTCTTGGGATGGG |
| bcl2 | | GTTCAGACAACTCTCCTCACTCTC | TTAGATTCAGACAGAGGGAGGAGT |
| siRNA | |  |  |
| siYBX1-1 | | GGAGUUUGAUGUUGUUGAATTUUCAACAACAUCAAACUCCTT | |
| siYBX1-2 | | GGUUCCCACCUUACUACAUTTAUGUAGUAAGGUGGGAACCTT | |
| siNEK6-1 | | AGAUGAUCAAGUACUUUAATTUUAAAGUACUUGAUCAUCUTT | |
| siNEK6-2 | | GACAGUUCAGCGAGGUGUATTUACACCUCGUGAACUGUCTT | |

Table S2 Clinical information of endometrial cancer samples

| Sample | Age | Pathologic type | FIGO stage | histological grade |
| --- | --- | --- | --- | --- |
| 1 | 52 | endometrioid adenocarcinoma | IA | G1 |
| 2 | 80 | endometrioid adenocarcinoma | IB | G3 |
| 3 | 73 | endometrioid adenocarcinoma | III | G1 |
| 4 | 56 | endometrioid adenocarcinoma | III | G2 |
| 5 | 49 | endometrioid adenocarcinoma | IA | G1 |
| 6 | 73 | endometrioid adenocarcinoma | IA | G1 |
| 7 | 47 | endometrioid adenocarcinoma | IA | G2 |
